# Using Network and Complexity Theories to Understand the Functionality of Referral Systems for Surgical Patients in Resource-Limited Settings, the Case of Malawi

**DOI:** 10.34172/ijhpm.2021.175

**Published:** 2021-12-22

**Authors:** Chiara Pittalis, Ruairí Brugha, Leon Bijlmakers, Frances Cunningham, Gerald Mwapasa, Morgane Clarke, Henk Broekhuizen, Martilord Ifeanyichi, Eric Borgstein, Jakub Gajewski

**Affiliations:** ^1^Department of Epidemiology and Public Health Medicine, Royal College of Surgeons in Ireland, Dublin, Ireland.; ^2^Department for Health Evidence, Radboud Institute for Health Sciences, Radboud University Medical Centre, Nijmegen, The Netherlands.; ^3^Wellbeing and Preventable Chronic Disease Division, Menzies School of Health Research, Brisbane, QLD, Australia.; ^4^College of Medicine, University of Malawi, Blantyre, Malawi.; ^5^Department of Health and Society, Wageningen University and Research, Wageningen, The Netherlands.; ^6^Institute of Global Surgery, Royal College of Surgeons in Ireland, Dublin, Ireland.

**Keywords:** Complexity Science, Network Analysis, Systems Thinking, Referrals, Surgery, Developing Countries

## Abstract

**Background:** A functionally effective referral system that links district level hospitals (DLHs) with referral hospitals (RHs) facilitates surgical patients getting timely access to specialist surgical expertise not available locally. Most published studies from low- and middle-income countries (LMICs) have examined only selected aspects of such referral systems, which are often fragmented. Inadequate understanding of their functionality leads to missed opportunities for improvements. This research aimed to investigate the functionality of the referral system for surgical patients in Malawi, a low-income country.

**Methods:** This study, conducted in 2017-2019, integrated principles from two theories. We used network theory to explore interprofessional relationships between DLHs and RHs at referral network, member (hospital) and community levels; and used principles from complex adaptive systems (CAS) theory to unpack the mechanisms of network dynamics. The study employed mixed-methods, specifically surveys (n=22 DLHs), interviews with clinicians (n=20), and a database of incoming referrals at two sentinel RHs over a six-month period.

**Results:** Obstacles to referral system functionality in Malawi included weaknesses in formal coordination structures, notably: unclear scope of practice of district surgical teams; lack of referral protocols; lack of referral communication standards; and misaligned organisational practices. Deficiencies in informal relationships included mistrust and uncollaborative operating environments, undermining coordination between DLHs and RHs. Poor system functionality adversely impacted the quality, efficiency and safety of patient referral-related care. Respondents identified aspects of the district-RH relationships, which could be leveraged to build more collaborative and productive inter-professional relationships in the future.

**Conclusion:** Multi-level interventions are needed to address failures at both ends of the referral pathway. This study captured new insights into longstanding problems in referral systems in resource-limited settings, contributing to a better understanding of how to build more functional systems to optimise the continuum and quality of surgical care for rural populations in similar settings.

## Background

**Key Messages**

** Implications for policy makers**

- Investing in improving the functionality of the inter-hospital referral system should be a high-priority in policy-makers’ agendas as it is critical for ensuring the efficiency, effectiveness and patient-responsiveness of surgical care delivery across care levels.
- An in-depth understanding of the complexity of functionality of the referral system is necessary to inform evidence-based interventions.
- Interventions must consider the dynamic interactions between the different elements of the system rather than focus on improving isolated components.
- The establishment/enhancement of structures to foster better coordination between district and referral facilities should be prioritised.

** Implications for the public**

The patient referral system, which enables a patient in need of specialised care to get from a district to a specialist hospital, is key to safe, timely and affordable surgical care for underserved populations in Africa. This mixed-methods study demonstrated that poor coordination, lack of guidelines and uncollaborative working relationships between surgical staff working at district and specialist hospitals can be detrimental. Staff training and health-system strengthening interventions are needed to improve the functionality of the referral system, so as to ensure an optimum continuum of care for surgical patients, especially those who live in rural and remote areas. Investments at facility level will not achieve maximum benefits if not designed to advance the functionality of the surgical referral system as a whole.

Conditions amenable to surgery are among the biggest causes of morbidity and mortality in low- and middle-income countries (LMICs), especially in sub-Saharan Africa (SSA) where an estimated 95% of the population has no access to surgical services.^1^ Furthermore, urban-rural inequalities persist: surgery – a proven and often life-saving intervention – is predominantly accessible to urban populations in many African countries, with only one surgeon per 2.5 million people in rural areas.^2^ Emerging evidence demonstrates that major surgeries can be undertaken safely and effectively at district level hospitals (DLHs), making them accessible to otherwise neglected rural populations.^3^ In recent years this has led to efforts to strengthen the surgical capacity of DLHs, based on the assumption that if these facilities are sufficiently staffed and equipped, they could perform a broader range and higher volume of operations, streamlining referrals to higher levels, reducing delays in care delivery and improving health outcomes.^3,4^

Expanding frontline capacity at district facilities must go hand-in-hand with adequate integration with specialist surgical services at higher level hospitals through the patient referral system. An effective and efficient referral system from district to specialist facilities is essential to save lives and ensure the continuum and quality of care by facilitating timely access to necessary services not available locally.^5,6^ But the referral process does not simply entail transferring patients from one hospital to another, nor does it end when patients are discharged from the referral hospital (RH).^5^ It also requires adequate training, protocols, proper communication and coordination between levels of care to ensure patient safety, as well as support (in person or remotely) from higher to lower levels to help manage patients at the lowest level of care when this can be done safely and effectively.^5^ As such, the relationships between district and RHs in the referral system are often multidimensional. Hospitals share responsibility for referred patients, but they also share information, knowledge and resources in a complex network of interactions.^7^ This involves different specialties, professions, organisational and managerial structures, across geographical locations, working together to enhance care delivery.

While it is widely known that in many LMICs referral systems across care levels are fragmented and do not function optimally,^4,8^ there is a scarcity of studies focused on identifying and analysing the interactions between actors in these systems and how they contribute to their functionality. A recent review of relevant literature found that existing research involving LMICs only addresses particular aspects of surgical referrals.^9^ Many studies measure referral rates and patterns, and list the reasons for referral,^10-13^ but they fail to explore the different dimensions of a referral system, the barriers and/or enablers for effective referral and communication and feedback mechanisms. Studying only selected aspects of the system in isolation is insufficient to understand the functionality of the system as a whole, which may lead to missed opportunities for improvements.

This paper intends to fill this knowledge gap. It builds on a previous study on patterns, quality and appropriateness of surgical referrals in Malawi,^14^ which identified and quantified some of the shortcomings in the referral system. The aim of this study is to undertake a deeper exploration of the referral system for surgical patients (hereinafter referred to as the “surgical referral system”) and unpack how the different aspects of the system, and the interacting behaviour of care providers within it, jointly contribute to its functionality. By taking a whole-system approach,^15^ it also seeks to unveil how the shortcomings described in the earlier work came about. Lessons from this research aim to inform the debate on the role of the referral system in expanding the delivery of safe, timely and affordable surgical care to underserved populations in SSA.^6,16^

## Methods

###  Methodological Approach

Through the referral system, healthcare organisations work interdependently in a network of multidimensional relationships to deliver a continuum of care to patients.^7^ The capabilities of the system rest on many interacting elements, consisting of^17^: the hardware of available resources (eg, infrastructure, staffing, equipment, funding); the tangible software of knowledge, skills and processes of decision making; and the intangible software of relationships, communication and values. The intangible features, in particular, are important in shaping the behaviours of providers in the system and its overall “power to perform.”^17^ Hence, the focus of this study is not just on the hardware, but rather on the interactions within and across hospitals, and how whole-system outcomes, such as the continuum of care, are generated collectively (ie, by service providers at DLHs and RHs).^7^

Measuring these dimensions required us to develop an analytical framework able to capture the complexity of these relationships. We integrated concepts from two systems-thinking approaches^18,19^: network theory, concerned with examining how elements within a system interact,^19^ and complexity theory, defined as ‘*a perspective that conceptualises relationships of components within a system as the foundation from which the properties of a system emerge*.’^20^ Our analytical approach involved three steps.

Firstly, we employed network theory to depict the structure upon which the network of relationships among hospitals in the Malawi surgical referral system is built.^18^ We adapted the model proposed by Cunningham et al,^21^ which investigates interprofessional relationships in health service networks at three levels – community, network and member levels – within the context of network characteristics and operating environment. The first dimension analyses the joint delivery of servicesby network members to the community, defined as the population served by the network.^22^ In our study we interpreted this as those surgical patients who benefit (or are expected to benefit) from the referral network. We aimed to capture the services normally expected to be provided through the referral network (ie, facilitating access to specialist care not available locally), as well as any other additional services hospitals can provide by being part of the network as opposed to working in isolation. The second dimension examines whether the network operates as a viable entity, in terms of connectedness between district and specialised hospital services and coordination. We considered the following aspects: extent of referral communication and consultation between district and RHs; continuous exchange of information and knowledge (including feedback); and management of patient transfer across facilities. The third dimension assesses whether being involved in the network is beneficial to its members (ie, district and RHs, and clinicians within them). In particular, we investigated any convergent and divergent behaviours among network members and examined how these affect every-day practice.

**Figure 1:**
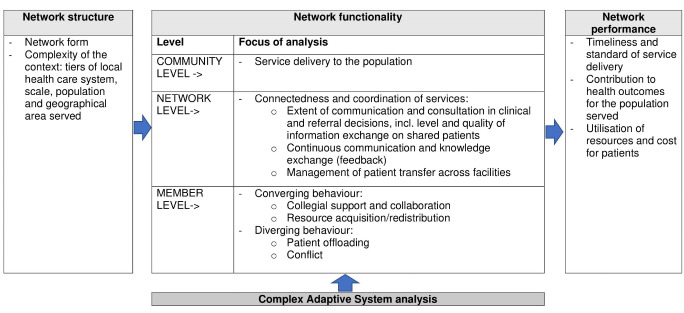
Conceptual Framework. Adapted from Cunningham et al.^21^

The adapted Cunningham et al model^21^ used in our study, and the list of parameters considered under each domain, is illustrated in Figure 1.

The multi-level relationships are not static, they change and evolve over time.^18^ Hence our second step (bottom of Figure 1) was to integrate the Cunningham et al model^21^ with key principles from the theory of complex adaptive systems (CAS) as a conceptual approach to unpack the mechanisms of network dynamics^19^ and deviations from the intended way of working. Many properties of CAS in healthcare have been described in the literature.^19^ For the purpose of our study we considered the following properties of CAS particularly relevant to understand the surgical referral system in Malawi:

- A CAS is a dynamic network of agents, constantly reacting to what the other agents are doing, which in turn influences behaviour and the network as a whole.^23^
- Hospitals are agents in the CAS, but each of them is also a CAS in itself, made up of individual agents such as managers, clinicians, patients and other stakeholders *(CAS are nested in other CAS).*^23^
- Agents’ behaviour may unfold in unanticipated ways depending on the circumstances and problems encountered, and because actions are highly interconnected, the action of each agent will change the context for the other agents in the system *(non-linear interdependencies).*^19^
- *Agents act autonomously, guided by internalised basic rules *which reflect their needs and desires, and their own understanding of the system, but are not homogeneous and may not necessarily be shared among all.^23^
- As they gain experience, *agents learn and adapt *their behaviour, constantly re-organising to cope with changing internal and external environmental demands (*self- organisation ).*^19,23^
- The overall system behaviour *emerges* from this dense pattern of interactions and over time *co-evolves* with the healthcare organisations and individuals which make up the whole.^19,23^

In the light of the considerable resource and operational challenges faced by the health sector in Malawi, these theoretic concepts are instrumental to understanding how the referral network self-organises to find the best fit to its environment and to determine its resilience.^19,24^ How agents connect and relate to one another is critical to the survival of the system.^23^

The third step (right side of Figure 1) was to determine how these multi-level sets of relationships, and the influence of external and contextual factors, affect the performance of the surgical referral system in terms of meeting the needs of the patients and their families, efficiency in utilisation of public resources and contribution to health outcomes.^21^

###  Study Setting

Malawi is a low-income country in SSA, with a population of about 17.5 million people in 2018^25^ and high levels of poverty (71% of the population live below the poverty line).^26^ The country is comprised of three administrative areas, the Northern, Central and Southern regions, and is densely populated, with 84% of the population based in rural areas.^25^

The public healthcare system has three tiers, linked to each other through an established vertical referral system.^27^ At the lowest level are primary level facilities, mostly responsible for promotive, preventive and basic curative healthcare. At secondary level are 24 public DLHs, each with a catchment area of between 140 000 and 1 400 000 people. Approximately 29% of all primary and secondary health services, especially maternal health, are provided by religious institutions organised under the Christian Health Association of Malawi, through a service agreement with the Ministry of Health. At the tertiary level there are four central hospitals (CHs), each with a catchment population of several millions.^27^ These facilities are located in the four largest cities and host all of the specialist surgical workforce in the country, which at 0.43 per 100 000 population is an extremely low density compared to international standards.^28^

The hospital system is hindered by resource scarcity, poorly developed financing mechanisms and weak governance,^27^ leaving a large unmet need for surgical care in the country.^29^ Financing and management of health resources at district level are controlled by a dedicated administrative authority, the District Health Office. DLHs have limited autonomy and decision-making space, with financial allocations that are often below needs.^30^ For patients, essential healthcare is free of charge at the point of entry but bypass fees have been introduced at the RHs to discourage patients circumventing lower facilities to seek treatment directly at higher levels.^27^

 DLHs are meant to provide for the basic and essential surgical needs of the population, and to refer more complex cases to RHs. Despite the existence of surgical graduate programmes, surgical services at DLHs are predominantly provided by generalists - Medical Officers and/or general non-physician clinicians. Good integration with specialist services at RHs is important for supporting these frontline health workers, who often deliver care in isolated rural settings with limited surgical training and supervision.

###  Sampling and Data Collection

This research, conducted in 2017-2019, employed mixed-methods and iterative data collection, reflecting the nature of inquiry in CAS research.^31^ The first round (2017) was undertaken as part of a situation analysis for the SURG-Africa research project.^32^ Data were collected to map the surgical referral network, and to conduct an initial assessment of referral practices and resource availability at DLHs. Information on the mandate, resources and governance of the referral network, as well as the intended role of its members, was gathered through a review of national health policies and other background documentation. A survey was administered in 22 of the 24 surgically active public DLHs country-wide (two DLHs were omitted due to inaccessibility at the time of data collection), and semi-structured interviews were carried out with members of the surgical team at a sample of nine DLHs (see Table 1).

**Table 1:** Characteristics of Surveyed District Level Hospitals

**Facilities (n = 22 DLHs)**	**Mean/DLH**	**SD**	**Min-Max**
Surgical team			
Surgical providers	17	6.42	9-31
Anaesthesia providers	2	0.66	1-4
Theatre nurses^a^	6	4.05	1-15
Bed capacity	261	67.31	174-456
Functional operating rooms	1	1.18	1-2
Functional vehicles^b^	4	1.79	1-7

Abbreviations: DLH, district level hospital; SD, standard deviation. ^a^This includes general nurses and specialised nurses. ^b^This refers to any vehicle available for the transport of patients, with or without medical equipment.

 Once key referral links were determined, we established a data collection system to capture patient flows across hospitals. Given resource limitations, we focused on the Southern region where SURG-Africa project coordination was based. Trained data collectors were stationed at the surgical units of the two main RHs for this region: Queen Elizabeth Central Hospital (QECH), the largest tertiary facility in the country, and Zomba Central Hospital (Zomba CH). Incoming surgical referrals at these two sentinel RHs were tracked during a six-month period (November 2017-April 2018 in QECH and December 2017-May 2018 in Zomba). Information collected concerned patient demographics and clinical information, referral documentation, details of referral and hospital of origin.

 This initial evidence informed the subsequent data collection. In early 2019 we repeated the survey of DLHs to gather further information on referral communication practices, including sharing of feedback. We then interviewed staff at RHs to gather their perspectives on the state of the surgical referral network, key obstacles, impact on their work and suggestions for improvements. At RHs, we purposively selected and interviewed personnel who were the first-point of contact for incoming referrals, identified through staff lists where available and through snowball sampling.

Ethical approvals for the study were obtained from all relevant authorities. All data collection was conducted in English. Surveys and interviews were administered in person by project researchers. Details of the tools used are described elsewhere.^32^ A summary of data sources and participants is provided in Table 2.

**Table 2:** Summary of Data Sources and Participants

**Data Sources**	**Timing**	**Sample**	**Participants**	**Focus Areas**
DLHs surveys	Jul-Sep 2017 and Mar 2019	22 DLHs	Surgical providers, anaesthesia providers and theatre nurses part of DLH surgical teams	Referral links across hospitals, referral practices (including communication and feedback) and resource availability at DLHs
Interviews with DLH staff	Jul-Sep 2017	12 interviews at 9 DLHs	Surgical providers, anaesthesia providers and theatre nurses part of DLH surgical teams	Key reasons for referrals and further details on referral practices, including unnecessary referrals
Database of incoming surgical cases received at the sentinel RHs	Nov-Dec 2017-Apr/May 2018	2 RHs	QECH, Zomba CH	Referral flows, including case details, type of referral, place of origin, transport mode and referral documentation
Interviews with RH staff	Oct 2018-Jul 2019	8 interviews at 3 RHs	Surgical interns, registrars, non-physician clinicians and the specialists supervising and coordinating them	Type of incoming referrals, appropriateness and quality of incoming referrals (communication, documentation, pre-referral management and stabilisation, transport), feedback practices at the RH

Abbreviations: QECH, Queen Elizabeth Central Hospital; Zomba CH, Zomba Central Hospital; DLHs, district level hospitals; RH, referral hospital.

###  Data Analysis

 Quantitative data were analysed using IBM SPSS v26. Where appropriate quantitative data sources were triangulated with each other and with qualitative data.

All interviews with key informants were audio recorded following informed consent from the participants and then transcribed. A thematic analysis of the interview data was conducted in NVivo v12, following a mixed top-down and bottom-up approach,^33^ guided by the framework in Figure 1. A first coding framework and structure of the analysis were drafted by the lead author and discussed with the wider team of researchers. These were then revised and finalised in a collaborative manner. The qualitative data analysis aimed to gather further details about the context in which the network operates and to provide insights into network dynamics from the perspective of its members and potential areas of improvement. Further details on the integration of data sources are provided in Supplementary file 1, Table S1.

## Results

To fully appreciate and potentially improve a complex system we must have a sufficiently good understanding of it,^34^ hence the presentation of results starts with a description of the Malawi surgical referral network structure (ie, the links between hospitals), followed by an assessment of how hospitals interact and determine what the network does, according to the model in Figure 1.

###  Network Structure

**Figure 2:**
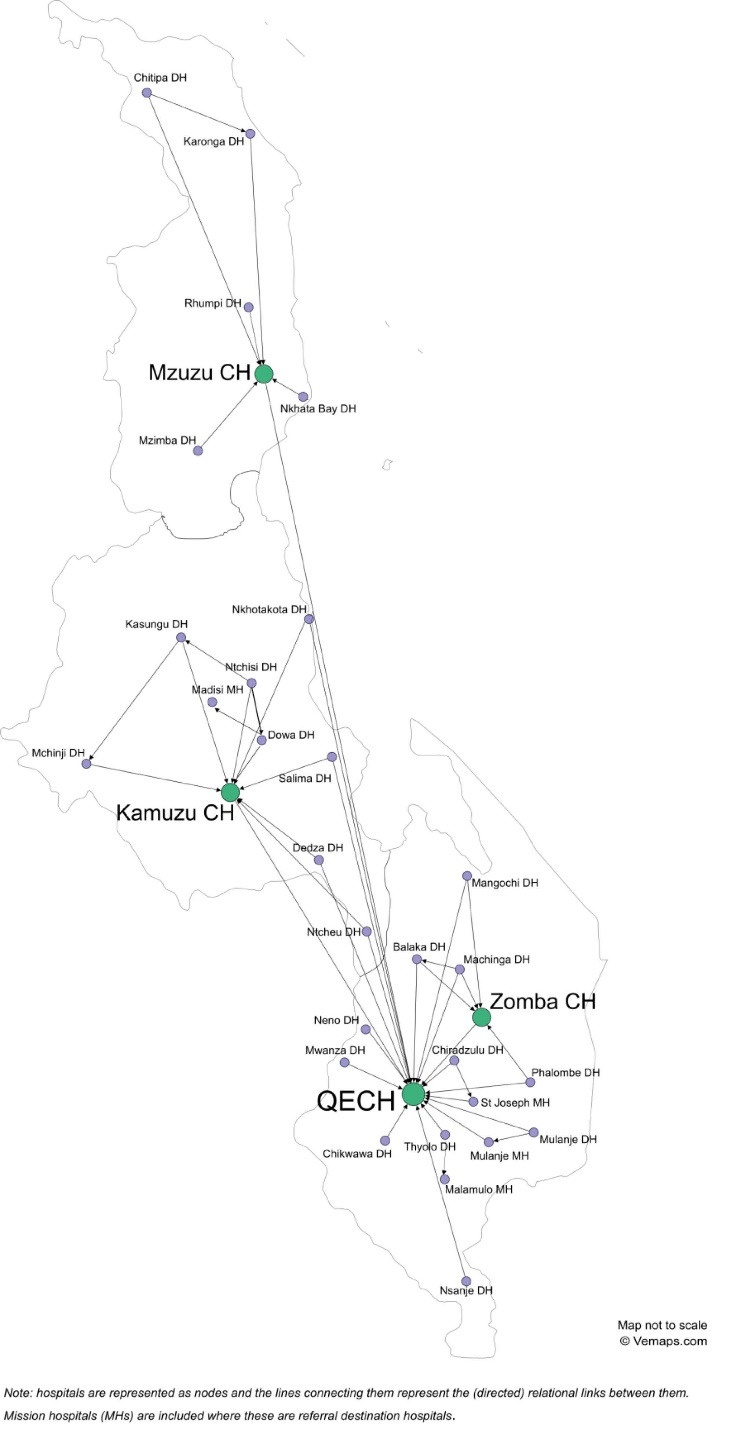
The Surgical Referral Network in Malawi. Abbreviations: QECH, Queen Elizabeth Central Hospital; Zomba CH, Zomba Central Hospital; Kamuzu CH, Kamuzu Central Hospital; DH, District Hospital.

The object of our study is a public health service delivery network (network form), with membership, mandate and geographical scope established by the Ministry of Health.^27^ The patterns of surgical referrals from DLHs to RHs observed in the network are represented by the unidirectional arrows in Figure 2, which is a georeferenced map of the Malawi surgical referral network including all public sector hospitals. The surgical patient referral pattern reflects the distribution of surgical specialists countrywide, rather than following the administrative boundaries of the three regions. In the Southern region QECH, better resourced than Zomba CH which has only one specialist surgeon, attracts almost all incoming referrals. It also receives referred cases from the other regions, notably from DLHs in the southern part of the Central region - see Figure 2 and Supplementary file 2, Table S2.

Referral links also exist between tertiary level hospitals, particularly used for the transfer of patients with congenital abnormalities and tumours from other CHs to QECH (see Supplementary file 2, Table S2 and S3). This is due to the fact that QECH employs almost all paediatric surgical specialists in Malawi and one of the only two oncologists. During the course of our study Zomba CH was in the process of establishing a urology centre. While the data reported in Supplementary file 2, Table S2 (December 2017- May 2018) precede this development, by the time we conducted the interviews in Zomba (June 2019) local staff confirmed that they had started to receive urology cases from other hospitals, including from as far as Kamuzu CH in the Central region.

Another deviation from the intended vertical flow of patients is the presence of horizontal referral links between pairs of district hospitals. These occurred in 46% (10/22) of sampled DLHs (see Supplementary file 2) and survey respondents usually attributed such referrals to resource shortages at some public sector DLHs.

###  Network Functionality

 We examined the functionality of the referral network as emerging from the dynamic interactions between DLHs and RHs at the community, network and member levels, and how these responded to their environment and each other. While we discuss these levels separately for presentation purposes, there is considerable overlap among them and they all contributed to the network’s overall service delivery patterns.

####  Community Level

At the community level, respondents to the survey (Supplementary file 2, Table S4) and the interviews reported that the referral network provides or enhances service delivery to the population in two ways. Firstly, it facilitates access to specialist advice or Intensive Care Unit care which are not available at district level, or diagnostic services only offered centrally, such as computerized tomography scans or magnetic resonance imaging. This is in line with the intended scope of the referral network. RH respondents reported that the fact that district surgical teams are able to recognise their skills limits and refer patients is beneficial for the patient and the functionality of the care system. A RH respondent provided the example of: “*some who are having headache for months and they [district clinicians] were thinking of malaria, treating malaria. But when they sent the patient to us, we viewed and we did a scan and found that it’s a brain tumor. So sending to us that patient with a brain tumour, it’s something very positive, because to them if they will stay with the patient, they will not assist the patient. They will just keep on wasting their time” *(26RMW).

 A second unintended but critical network function reported by respondents is to enable DLHs to mitigate problems affecting their capacity to perform surgery and to ensure continuity in services. When blood is not available, or failure of major equipment or infrastructure (eg, electricity blackouts or interruption of water supply which are frequent in Malawi) occurs, the network allows the transfer of patients to other facilities that are better equipped.

Some respondents reported that the referral network also offers clinicians a way to manage expectations from the community. As described by a respondent, patients’ wishes and preferences play a role in referral decisions: *“Some of them, they do ask maybe: ‘my closest [hospital] to home is Zomba. So, can you just help me, like write me to be referred to Zomba’” *(28RMW).

####  Network Level

Another key dimension of referral network functionality, as depicted in Figure 1, is connectedness and coordination between sending and receiving facilities through two-way exchange of information and coordinated management of patient transfer. Upward flow of information consists of consultation between DLHs and specialists at RHs on whether referral of a patient is required, how to handle the transfer, and sharing of essential patient information. At the receiving end this allows the RH to monitor and prepare for incoming referrals. Downward flow of patient information from the RH specialist to the referring DLH clinician is important for any necessary post-discharge care, as well as for sharing feedback on the referral.

####  Referral Communication and Consultation

Almost all (96%, 21/22) surveyed DLHs (self-)reported routinely engaging in communication and consultation with RHs in their referral decisions, through instant messaging (32%, 7/22), phone calls (14%, 3/22) or a combination of the two (50%, 11/22). Interview respondents at RHs, however, highlighted that communication is not always sufficient or efficient. They noted that efforts vary from hospital to hospital, with particular facilities considered less engaged than others:* “they will just send the patient. Then when you call them back, they won’t pick up the phone. There are a few people there who I don’t think they understand the whole thing” *(27RMW).

A written communication tool is the referral letter, which is a standard form for clinicians to share essential information on the condition, results of investigations and treatment of shared patients. As shown in Supplementary file 2, Table S5, almost one third of incoming referrals at the sentinel RHs arrive with no documentation. Over half of the completed letters do not state reasons for the referral and 21% (46/224) are not signed by the referring clinician.

Interview respondents at RHs added that the incomplete or insufficient information provided in the letters also extends to vital signs, investigations and treatment provided at the DLHs. They also explained that the lack of signatures prevents them from calling the referring clinician when clarifications on patient management are needed. The issue of incomplete information in referral letters was linked by a RH interviewee with DLH clinicians’ possible fear of reprimand: *“Sometimes maybe they are in a rush or they don’t want to expose themselves for the reasons why they referred the patient” *(28RMW).

####  Continuous Communication and Information Exchange (Feedback)

 The majority of surveyed DLHs (64%, 14/22) reported receiving only occasional or no feedback from RHs after patients have been referred. Interview respondents at RHs stated this is not part of standard practice:

“*I have never been told to give feedback. If anything, just to get more information and tell them that they send inadequate referral or that kind of thing. [...] If they ask me to give feedback, I use my own unit [phone credit], and how long can I speak?” *(24RMW).

Another reported deterrent to the provision of feedback is that it is commonly perceived as a way to police and reprimand DLH staff. As explained by a RH clinician feedback is *“minimal because we didn’t even know who we were talking to. They will tell you, ‘Oh, I was not the one.’ ‘But somebody put your number there!’ [...] So they will try to dodge the bullet” *(27RMW).

 The only formal provision in place is a dedicated space in the referral letter for writing feedback. Interview respondents explained that even when filled in, patients usually return to their homes after discharge from the RH so these documents are usually lost to the system and feedback relies on the initiative of the more proactive clinicians who follow up via phone.

####  Management of Patient Transfer

 Another major issue affecting referral coordination between sending and receiving facilities reported by respondents is the scarcity of adequately equipped ambulances at DLHs and limited fuel for the transport of patients to higher levels. A common strategy adopted by DLHs to balance meeting patients’ needs with the constraint of transport capacity is to pool patients in ambulances: patients who require referral for elective surgery are transported when emergency cases are being referred, medical patients are mixed with surgical patients in the same ambulance trip, and the transfer of patients may be coupled with other tasks (eg, transporting corpses). As explained by interviewees, these practices are helpful for DLHs but it means that, unless there is a life-threatening emergency, ambulances tend to leave the DLHs late in the day, once they reach full capacity.

 The same considerations apply to choices regarding staff accompanying patients in the ambulance. RH respondents noticed that DLHs further away tend to send escorts since the long journey increases risk for patients, while closer facilities send junior nurses or no accompanying staff at all. However, due to the pooling of different types of patients, respondents felt that even when escorts are present they are insufficient compared to the number of patients in the ambulance.

####  Member Level

In CAS theory individual behaviours are strongly linked with the beliefs, values and priorities of individual healthcare organisations, as well as the agents within them, that make up the system. Beliefs, values and priorities are heterogeneous and can converge or diverge at the same time, depending on the perspective.^19^ Our findings demonstrate that when beliefs, values and priorities among members of the referral network converge, this fosters cooperative behaviours which are beneficial for individuals and the network as a whole. When values, beliefs and priorities diverge, self-interest prevails to the benefit of individual clinicians but impacting negatively on other clinicians and potentially on patients. Examples are presented below.

####  Converging Behaviour

Both qualitative and quantitative findings reveal that members of the network have a shared understanding that shortages of all types of resources are deep-rooted features of the working environment in Malawi,^27^ impeding their ability to provide optimal care for patients. Respondents reported that ensuring service delivery in these settings may require a flexible approach towards clinical guidelines. For example, RHs clinicians stated that while they may not always understand or know the exact reasons behind the referral decisions of sending hospitals, they prioritise what is best for the patient: *“because they [the sending hospitals] are already disadvantaged, we don’t have anything to say, we just accept it, proceed with management” *(22RMW).

####  a. Collegial Support and Collaboration

Interview respondents highlighted that particular DLHs have established positive working relationships with RHs over the years to the benefit of both parties. This is often driven by the spirit of initiative of individual district clinicians, who have invested time and efforts in cultivating collaborative relationships with RH specialists, and persistence to maintain them. As explained by a RH respondent when describing one such clinician: *“he is very collegial. All the time, if [name of district clinician] sends something [a case] you know for sure that’s something which he really needed help with.[...] If [he] was behaving like all the other ones he would have been a tough guy, giving us all the problems, but he is very professional and he communicates very well” *(27RMW).

####  b. Resource Acquisition and Redistribution

 Individual DLHs also use the referral network to exchange resources and cope with challenging situations. For example, respondents reported that Mulanje DLH refers patients to the nearby Mulanje mission hospital in case of (frequent) shortage of essential resources, but occasionally also “borrows” the mission hospital’s more experienced clinicians to manage particularly difficult cases locally and avoid referral. A number of other DLHs reported having informal local arrangements with each other allowing them to move their whole surgical team to perform urgent operations at nearby facilities when there are major failures in equipment or interruptions to electricity or water supply. Others share supplies and other surgical essentials, such as instrument sets or gowns.

####  Diverging Behaviour

####  a. Patient Offloading

 An evident example of this in our data is when district clinicians use the referral network to shy away from their responsibility for the care of patients with simple surgical conditions that could have been managed locally, shifting that responsibility to RHs. 73% (16/22) of surveyed DLHs reported these unnecessary referrals among their staff.

**Figure 3:**
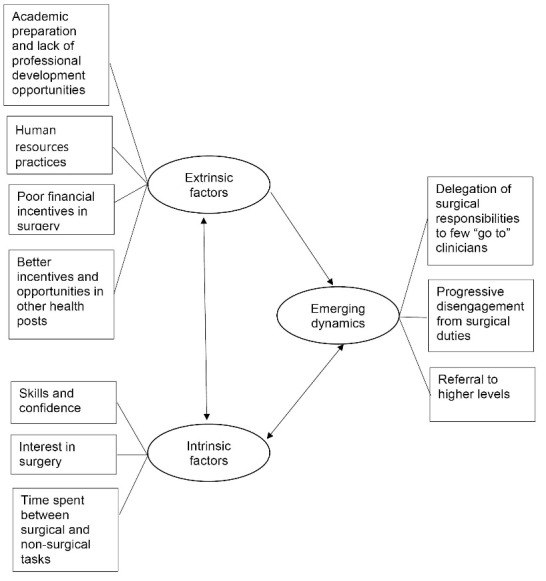
Factors Influencing District Clinicians’ Attitudes Towards Surgical Duties as Reported by Interview Respondents.

As described by Franco et al, workers’ willingness to fulfil their tasks and exert an effort towards shared goals depend on the combination of extrinsic (health sector policies, organisational practices, working environment) and intrinsic factors (eg, personal values, job expectations, self-esteem).^35^ Our qualitative analysis uncovered the factors at play at Malawian DLHs, illustrated in Figure 3 (further details in Supplementary file 2, Table S6).

The interplay of these factors affects district clinicians’ commitment to and engagement in surgical duties. As reported by interview respondents, many district clinicians shy away from surgery in favour of other less onerous and more financially rewarding tasks, leaving the bulk of surgical service delivery to a few of the more surgically experienced and committed staff. Over time this creates a disconnection between the “go to” persons for surgery and the other DLH staff, and hinders the creation of fully functional district surgical teams where members are able to support and cover for each other when needed. A district clinician described this situation at his hospital: *“as of now it’s a problem because there is a big [circumcision] campaign, so on the ground we have very few staff for surgeries, [...] who are qualified but they are not very good persons to do the procedures” *(10DMW).

Respondents explained the result is that at night and weekends when hospitals operate with reduced staff, or whenever the more skilled provider is not available, gaps in service provision inevitably appear. Clinicians, faced with uncertainty or increased workloads, choose to refer patients to offload responsibility for the patient’s care, either temporarily or permanently, to the next care level. As described by a RH respondent: *“somehow there is always a laissez-faire sort of attitude, to give an excuse [...]. But is that true? No. I am just doing it so that maybe I need to go somewhere else to do something. You know, that kind of thing. In the end, you have failed to provide health services” *(24RMW).

####  b. Conflict

Over time these practices have built up frustration, leading to deteriorating relationships across hospitals and conflict, with certain hospitals developing a negative reputation among their peers. In response RH clinicians have developed various informal strategies to deal with the issue. Some have learnt to distinguish signs of potential unnecessary referral and mislabelled cases: *“There is always a clue because normally the person will put a signature, just a signature, and the name will be written so illegible. So I always know to say ‘this patient, I have to review them properly’” *(24RMW).

Others use the acumen of more experienced nurses and clinicians triaging patients on arrival to identify unnecessary referrals that can safely be sent back to the originating hospitals immediately without admission. Examples may be simple cases such as dislocations or epididymitis. As justified by a RH respondent, for these patients there is *“nothing surgical that you are going to do. And you are also preventing crowding, besides preventing them to get concomitant infections in the hospital. They [the RH staff] are also taking all that they can manage to take on, so you have to say ‘ok, we send them home’” *(24RMW).

###  Network Performance

 Collaborative relationships between DLHs and RHs around a shared objective of providing optimal patient care are of great importance in determining the referral network’s overall performance, as the components of the system are mutually reliant on each other and share reciprocal relationships. As described in the previous sections, however, DLHs and RHs do not always work in unison, with a number of implications.

####  Care Delivery Timeliness, Standards and Impact on Health Outcomes

Respondents reported that the tendency of certain DLHs to refer at particular times or only when ambulances are at full capacity causes a delay in transfer to the RH, with potential deterioration of the patient’s clinical status. In turn, this affects the effectiveness of treatment and clinical outcomes at the receiving end: “*during Monday, Tuesday, Wednesday, especially on Wednesday, we now see cases that have overstayed at the district. So we have someone with perforation, a clear perforation - they [the DLH] did X-Ray on admission and they saw that it was perforation. They should have sent to us. And when they send to us then we have to stabilise the patient. Some die, some survive” *(24RMW).

Similarly, when patients are transferred without adequate escorts their safety may be compromised during the journey:* “if they are just sent with a driver, most of the time they come here dry and we need to resuscitate them two, three hours before we can even operate” *(27RMW).

Lack of referral coordination and insufficient communication also cause delays and affects care at the receiving end. RH clinicians reported often having to handle incoming referrals “*starting from zero”* rather than as part of a continuum of care, with unnecessary duplication of effort. In very urgent cases, this may also oblige RHs to take risks: *“If we are not satisfied, we text them or call them through our switchboard to give us the details. Though most of them, they just say ‘we will send you’ and they don’t send us. So, at times we just enter the surgical theatre blindly, without knowing exactly what took place and that also affects our management. [...] At times you end up having something that you did not expect, so if the instruments are not there you are in trouble” *(22RMW).

Furthermore, at RHs incoming referrals add to the waiting list of operating cases, with emergencies taking priority. As explained by respondents, the unnecessary referral of simple cases that could have been handled at district level delays the care of complex cases that genuinely need specialist attention but may be less urgent. Multiple arrivals late in the day are particularly problematic for RHs because at night they have reduced staff numbers. This contributes to staff fatigue and may affect care standards, especially under the pressure of emergency cases. As explained by a RH clinician* “the patients come in at night and there is a whole bunch of them. At night, people tend to become less active and sometimes you are not thinking as well, because when a patient comes at 1am; the way you would think at 1am and 1pm are sort of different” *(25RMW).

####  Utilisation of Resources and Cost for Patients

 Referral practices emerging from our data affect the efficient use of resources in the health system and create additional costs for patients and their families.

Unnecessary transfers contribute to further draining the limited budget set aside for referral services. RH respondents stated that unnecessary referrals, the accumulation of incoming referrals in the evenings and having to manage additional referrals from outside their catchment area, deplete resources at the RHs and undermine their service delivery capacity: *“For example they come, they need blood. The blood, we have already exhausted; it means we are not going to transfuse them. It means we should wait for the National Blood Transfusion Service to bring some more blood. So it means those people are going to be mismanaged” *(22RMW).

From the patients’ perspective, transfer to higher levels rather than local treatment increases the financial burden on the household, especially low-income ones,^36,37^ as explained by a clinician: *“in Malawi or Africa we have a big community. If I’m sick then my uncle, my dearest friend, everyone is going to come just to demonstrate that we love each other. Now, for them to come here, to feed, to dress, to wash - all sorts of stuff, it is costly. So we admit someone with merely constipation that we could have done anywhere. [...] I find it very much costly” *(24RMW).

 Furthermore, as reported by respondents generally the DLH ambulance service is not intended to provide for the return home of the patient, so families have to foot the bill for the transport of the patient back home. This is particularly an issue where there is unnecessary referral of patients with low complexity conditions who are not deemed suitable for admission by the RH.

## Discussion

Our analysis identified a number of obstacles to the functionality of the surgical referral system in Malawi (eg, resource shortages, weak integration and inadequate coordination between sending and receiving hospitals) that have been previously reported across LMICs.^9^ The added value of this paper, we contend, is that the application of principles from network^21^ and complexity^19^ sciences in interpreting our findings has helped to reframe the conceptualisation of referral system functionality to reveal new insights into known problems. In addition, the picture of the surgical referral system emerging from our findings exhibits many characteristics of a CAS.

We showed that system functionality depends on a wide variety of interacting elements and is inextricably tied to the relationships between DLHs and RHs, as well as agents (surgical clinicians and specialists) within them. Because this is a public service delivery network, each agent has a specific role, with associated expectations and norms of behaviour. The Malawi Health Sector Strategic Plan^27^ broadly describes the distribution of services across care levels, but there is no detailed guidance on what surgical procedures should be done at district level and no clear transfer protocols for surgery, leaving room for interpretation and a certain degree of freedom to deviate from the intended way of working.

It is also important to emphasise that the system is ultimately made up of people, who make decisions under real-world conditions and their actions depend on their individual way of processing information and making sense of the environment around them.^19^ The result is that modest system alterations continuously take place as those on the frontlines of care subtly alter their practices or priorities in response to each other and the environment.^19^ We can see examples of this in our findings, for instance, in the progressive adaptation of referral patterns to changes in the distribution of specialists across the Southern region and countrywide, often disregarding administrative boundaries.

 Flexibility and adaptability also permit agents to act in discretionary ways that may improve, but can sometimes hinder, system functionality. Positive examples of these dynamics are the different ways in which hospitals work together to navigate the resource challenges frequent in Malawi. On the other side, there can be poor alignment among clinicians and healthcare organisations, as evident from the unnecessary referrals, inadequate communication and uncoordinated referrals documented in our findings. In which case, surgical referral system functionality deteriorates (patients in need of specialist surgical care are not appropriately referred in a timely way and with accurate information), adversely impacting quality, efficiency and safety of care.

Harnessing these synergies while mitigating dysfunctional behaviours is critical to enable the system to evolve in accordance with its purpose and to enhance its resilience.^19,24^ The referral network’s overall performance, and ability to cope with challenges in the environment, is the result of the many decisions made constantly by individual agents.^23^ It requires that those involved understand and actively work towards the common purpose of providing optimal care to patients in need of referral,^7,38^ for which high quality communication and coordination are essential.^38,39^ Studies of care dynamics demonstrate this requires acting on both the formal^40^ and informal (relational) structures aimed to foster coordination.^39^

In Malawi, weaknesses in the formal coordination structure identified by our study relate to the unclear scope of practice of district surgical teams and the lack of protocols to guide their work, for example regarding which surgical cases they should undertake and which ones they should refer to RHs; lack of standards on referral communication (including feedback) between DLHs and RHs; and misaligned organisational practices. For example, as described in our findings and in more detail elsewhere,^36^ inefficiencies in district budgetary allocations encourage DLH management to resort to various cost saving strategies, including patient pooling and limited use of escorts during referral. These practices have a negative knock-on effect on service delivery at the RHs, especially at night. More evidence-based resource allocation in the districts and/or more strategic organisational planning at RHs may need to be considered to improve coordination across care levels and to address system failures at both the sending and receiving ends of referrals. Standard unified procedures for referrals (including in relation to handling of elective vs. emergency transfers, presence and duties of escorts, logistics of transfer) should also be developed, tested and made available countrywide.

In regard to informal (relational) coordination, the main finding of our research is that the operating environment within the hospital sector is not always conducive to collaborative work, and is permeated by mistrust between DLHs (afraid of reprimand) and RHs (fearing opportunistic behaviour by district clinicians). Blaming an individual hospital or clinician, and ignoring the systemic picture, will not improve the system. The combination of technical, organisational and human weaknesses in the referral system play a central role in the development of these patterns^41^; and when discretionary decisions occur, as shown in our findings, there are usually underlying factors such as lack of confidence and professional support, pressing workloads and resource constraints. Hence, addressing these challenges will require interventions at multiple levels.

Firstly, there is a need for interventions to improve district clinicians’ commitment and teamwork in surgery, including to build their confidence, to provide better incentives and more efficient utilisation of the skill mix. Secondly, there is a need for interventions to improve mutual understanding and trust. This process could be facilitated by on-the-ground leadership from a mutually respected party,^38^ who could share their experience and assist as mediators of process change.^40^ This leadership could come from individual district clinicians who already have positive working relationships with RH specialists (and are usually the more experienced surgical providers at their hospital), and from committed surgical specialists, who in the highly hierarchical structure of the surgical profession are usually perceived and respected as figures of leadership.

An important suggestion made by our study participants, in line with both network^38^ and CAS^19^ literature, is to encourage more regular communication and interaction, creating opportunities for clinicians to look beyond their immediate surroundings, to learn about how their actions impact the work of others, and collectively find solutions to the complexities of their operating environment.^40^ More meaningful communications could be achieved via regular communication channels or, as proposed by interview respondents, by periodic in-person meetings bringing together specialists and district clinicians, or mentorship visits to the districts. This information has been used to inform the development of an intervention to strengthen referral services in Malawi, under the SURG-Africa project,^32^ of which this study was part of.

In conclusion, our study provided a comprehensive assessment of the functionality of the surgical referral system in Malawi, identifying several inefficiencies but also uncovering aspects of the district-specialist hospital relationships which could be used to develop more collaborative and productive relationships in the future. Despite the obvious need for investments in system inputs such as staff skills, facility infrastructure and resources, we share the view of other studies^42^ that, even with less than adequate resources, a public service network such as the referral network may perform if given a chance to learn how best to apply its limited resources. This is important for Malawi, as other LMICs, where resource shortages will likely persist in the foreseeable future.

###  Limitations

 Our study had two main limitations. Firstly, we only collected referral data at RHs. Lack of standard referral record systems at DLHs, the presence of multiple channels of outward referrals, and the high number of facilities, made logistics of data collection at the sending end impractical. While this is a limitation to the study, this choice ensured the creation of a smaller but more reliable dataset. For logistical reasons the selected RHs were in the Southern Region, where the SURG-Africa project was situated. Studies in Malawi’s other two regions might identify regional differences in referral flows. However, our survey and interview data confirmed that referral practices of sending hospitals (in terms of communication, pre-referral management etc) were similar, regardless of destination of the transfer.

 Secondly, it was beyond the scope of our study to interview other stakeholders in the referral system, such as hospital managers, patients and relevant authorities. Further research is needed to explore their perspective and potential additional lessons on the functionality of the referral system.

## Ethical issues

 Ethical approval was received from the Research Ethics Committee of the Royal College of Surgeons in Ireland, the project consortium lead (approval No. REC1417, REC1493) and the College of Medicine Research Ethics Committee in Malawi (approval No. P.05/17/2179, P.01/18/2336).

## Competing interests

 Authors declare that they have no competing interests.

## Authors’ contributions

 CP, JG, RB, and FC contributed to the conception and design of the study. EB, RB, JG, and LB obtained funding, CP and GM provided administrative and technical support. CP, JG, GM, and MC were responsible for acquisition of data. CP wrote the first draft of the manuscript under JG and RB supervision, and JG, RB, and LB contributed to its subsequent iterations. All authors contributed to analysis and interpretation of data, and critical revision of the manuscript for important intellectual content. All authors read and approved the final version.

## Funding

 This work was undertaken as part of the Scaling up Safe Surgery for District and Rural Populations in Africa (SURG-Africa) project, which is funded by the European Union Horizon 2020 Programme for Research and Innovation, under grant agreement No. 733391.

## Supplementary files

Supplementary file 1 contains Tables S1.

Supplementary file 2 contains Tables S2-S6.
