# Supplementary material for: Using Network and Complexity Theories to Understand the Functionality of Referral Systems for Surgical Patients in Resource-Limited Settings, the Case of Malawi
Source: Int J Health Policy Manag. 2021 Dec 22;11(11):2502–13. doi: 10.34172/ijhpm.2021.175 (PMC9818113; doi:10.34172/ijhpm.2021.175)
Supplement: Supplementary file 1 — contains Tables S1. [file ijhpm-11-2502-s001.pdf]

Article title: Using Network and Complexity Theories to Understand the Functionality of Referral Systems for Surgical Patients in Resource-Limited Settings, the Case of Malawi

Journal name: International Journal of Health Policy and Management (IJHPM)

Authors' information: Chiara Pittalis[1]*, Ruairí Brugha[1], Leon Bijlmakers[2], Frances Cunningham[3], Gerald Mwapasa[4], Morgane Clarke[1], Henk Broekhuizen[5], Martilord Ifeanyichi[2], Eric Borgstein[4], Jakub Gajewski[6]

[1]Department of Epidemiology and Public Health Medicine, Royal College of Surgeons in Ireland, Dublin, Ireland.

[2]Department for Health Evidence, Radboud Institute for Health Sciences, Radboud University Medical Centre, Nijmegen, The Netherlands.

[3]Wellbeing and Preventable Chronic Disease Division, Menzies School of Health Research, Brisbane, QLD, Australia.

[4]College of Medicine, University of Malawi, Blantyre, Malawi.

[5]Department of Health and Society, Wageningen University and Research, Wageningen, The Netherlands.

[6]Institute of Global Surgery, Royal College of Surgeons in Ireland, Dublin, Ireland

(*Corresponding author: chiarapittalis@rcsi.ie)

Supplementary file 1. Data Sources and Integration

Table S1: Data sources and integration

Network dimensions	Focus of analysis	Data sources
**Structure**	Network form	Relevant government documents and literature
	Complexity of the context	Relevant government documents and literature
	Georeferenced surgical referral patterns	DLHs first survey<br>Referrals dataset at sentinel RHs
**Functionality**		
Community level	Service delivery to the population	DLHs first survey<br>DLHs interviews and RHs interviews
Network level	Connectedness and coordination of services	
	- Extent of communication and consultation in clinical and referral decision making, including level and quality of information exchange on shared patients	DLHs second survey<br>RHs interviews<br>Referrals dataset at sentinel RHs (for the analysis of referral letters)
	- Continuous communication and knowledge exchange (feedback)	DLHs second survey<br>RHs interviews
	- Management of patient transfer across facilities	DLH second survey<br>RHs interviews
Participant level	Converging behaviour	
	- Collegial support and collaboration	DLHs first survey<br>DLHs and RHs interviews
	- Resource acquisition	DLHs first survey<br>DLHs and RHs interviews
	Diverging behaviour	
	- Patient offloading	DLHs first survey<br>DLHs and RHs interviews
	- Conflict	RHs interviews

**Complex Adaptive System analysis**		DLHs first survey<br>DLHs interviews and RHs interviews
**Performance**	Timeliness and standard of service delivery	DLHs interviews and RHs interviews
	Contribution to health outcomes for the population served	DLHs interviews and RHs interviews
	Utilisation of resources and cost for patients	DLHs interviews and RHs interviews

Data collection was jointly conducted by at least one international and one Malawian researcher at all times to minimise the risk of power imbalances. The collection of referral data at the sentinel RHs was done using local data collectors. Overall findings from the multiple data collection tools employed in this study were validated in three ways. Firstly in the DLH surveys, at least two members of the surgical team at each district hospital were present during the administration of the questionnaire to minimise potential recollection bias. These usually consisted of the theatre in charge and another surgical team member (surgical provider, anaesthesia provider or theatre nurse). Each question was read aloud to them by the researchers, and they were asked to discuss the answer and agree on the final response. Secondly, some of the questions were asked again during the DLH semi-structured interviews, giving respondents the opportunity to elaborate further on the answers provided in the survey. Thirdly, DLH survey and interview responses were triangulated with interviews with key informants at the referral facilities and data cross-checked across all databases (e.g. destination of referrals reported by DLHs in the survey vs. actual referral patterns documented in the database at the sentinel RHs). The interpretation of findings presented in this manuscript is the result of long-lasting discussion between all researchers involved – most of whom have long-standing experience working in Sub-Saharan Africa and deep knowledge of the district surgical system. The input of the Malawian researchers was also key in ensuring the interpretation of findings was in line with the situation on the ground.
