# Supplementary material for: Using Network and Complexity Theories to Understand the Functionality of Referral Systems for Surgical Patients in Resource-Limited Settings, the Case of Malawi
Source: Int J Health Policy Manag. 2021 Dec 22;11(11):2502–13. doi: 10.34172/ijhpm.2021.175 (PMC9818113; doi:10.34172/ijhpm.2021.175)
Supplement: Supplementary file 2 — contains Tables S2-S6. [file ijhpm-11-2502-s002.pdf]

Article title: Using Network and Complexity Theories to Understand the Functionality of Referral Systems for Surgical Patients in Resource-Limited Settings, the Case of Malawi

Journal name: International Journal of Health Policy and Management (IJHPM)

Authors' information: Chiara Pittalis[1]*, Ruairí Brugha[1], Leon Bijlmakers[2], Frances Cunningham[3], Gerald Mwapasa[4], Morgane Clarke[1], Henk Broekhuizen[5], Martilord Ifeanyichi[2], Eric Borgstein[4], Jakub Gajewski[6]

[1]Department of Epidemiology and Public Health Medicine, Royal College of Surgeons in Ireland, Dublin, Ireland.

[2]Department for Health Evidence, Radboud Institute for Health Sciences, Radboud University Medical Centre, Nijmegen, The Netherlands.

[3]Wellbeing and Preventable Chronic Disease Division, Menzies School of Health Research, Brisbane, QLD, Australia.

[4]College of Medicine, University of Malawi, Blantyre, Malawi.

[5]Department of Health and Society, Wageningen University and Research, Wageningen, The Netherlands.

[6]Institute of Global Surgery, Royal College of Surgeons in Ireland, Dublin, Ireland

(*Corresponding author: chiarapittalis@rcsi.ie)

Supplementary file 2. Additional Data

Table S2. Volume and types of incoming surgical referrals received at the sentinel RHs over the six months period Nov/Dec 2017 - Apr/May 2018

	**QECH** N(%)	**Zomba CH** N(%)
Total number of incoming referrals	269	72
… of which from outside regional boundaries	42 (15.6%)	0 (0%)
Hospital of origin type:		
Public	253 (94.1%)	72 (100%)
Mission	16 (5.9%)	0 (0%)
Hospital of origin level:		
District	224 (83.3%)	72 (100%)
Central	45 (16.7%)	0 (0%)
Type of surgical condition:		
Burns	25 (9.3%)	0 (0%)
Congenital abnormalities	100 (37.2%)	14 (19.4%)
Gastrointestinal conditions	17 (6.3%)	34 (47.2%)
Obstetric and gynaecological conditions	26 (9.7%)	1 (1.4%)
Trauma and orthopaedics	30 (11.2%)	2 (2.8%)
Tumours	21 (7.8%)	1 (1.4%)
Urological conditions	9 (3.3%)	1 (1.4%)
Others	41 (15.2%)	19 (26.4%)

Table S3. Breakdown of surgical referrals transferred to QECH over the six months period Nov 2017 - Apr 2018 by sending facility level and type of surgical condition

N(%)	**From district facilities (n=224)**	**From tertiary facilities (n=45)**	**Total (n=269)**
Burns	24 (10.7%)	1 (2.2%)	25 (9.3%)
Congenital abnormalities	80 (35.7%)	20 (44.4%)	100 (37.2%)
Gastrointestinal conditions	16 (7.1%)	1 (2.2%)	17 (6.3%)
Obstetric and gynaecological conditions	22 (9.8%)	4 (8.9%)	26 (9.7%)
Trauma and orthopaedics	29 (12.9%)	1 (2.2%)	30 (11.2%
Tumours	12 (5.4%)	9 (20%)	21 (7.8%)
Urological conditions	7 (3.1%)	2 (4.4%)	9 (3.3%)
Others	34 (15.2%)	7 (15.6%)	41 (15.2%)

Table S4. Key reasons for referral reported by surveyed DLHs (n=22)

**Reasons for referral**		N(%)*
Advanced care:	Access to specialist care	7 (31.8%)
	Access to post-op care/ICU	12 (54.5%)
Lack of capacity:	Lack of adequate skills**	16 (75.7%)
Lack of resources:	Lack of equipment and supplies	10 (45.5%)
	Shortage of blood	7 (31.8%)
	Infrastructure issues	6 (27.3%)

**Multiple responses possible*
***Lack of expertise in particular surgical procedures and/or lack of adequate skills when the few more experienced providers are not available*

Table S5. Details of referral letters accompanying incoming referrals at the sentinel RHs over the six months period Nov/Dec 2017 - Apr/May 2018

	**QECH** Tot referrals = 269<br><br>N(%)	**Zomba CH** Tot referrals = 72<br><br>N(%)	**Total** Tot referrals = 341<br><br>N(%)
Number of incoming referrals with referral letter	167 (62.1% of total)	57 (79.2% of total)	224 (65.7% of total)
Stated reasons for referral:			
Advanced care (access to particular specialist or diagnostic services)	59 (35.3%)	24 (42.1%)	83 (37.1%)
Need of ICU	3 (1.8%)	0 (0%)	3 (1.3%)
Lack of capacity	14 (8.4%)	1 (1.8%)	15 (6.7%)
Lack of resources	5 (3%)	0 (0%)	5 (2.2%)
General comment *'for further management'* with no details	79 (47.3%)	32 (56.1%)	111 (49.6%)
No reason given	7 (4.2%)	0 (%)	7 (3.1%)
Letters signed by sending clinician	147 (88%)	31 (54.4%)	178 (79.5%)

Table S6. Factors influencing district clinicians' attitudes towards surgical duties as reported by interview respondents

	**SAMPLE QUOTES**
EXTRINSIC FACTORS	
Academic preparation	*Sometimes we refer because other skills are not available […] because they are general clinical officers, they are not specialised in surgery. (03MDW)*
Lack of in-service training and opportunities to perfectionate surgical skills in the districts	*Once they learn how to do the caesarean sections they stop there. […] There is no that opportunity to say let's train them, no. (01DMW)*
Poor financial incentives in surgery	*People have been complaining about the allowances, they are not being paid. When you call them, they are not ready to come because of the way the management is handling things. (02DMW)*
Better opportunities and incentives in other posts	*Surgery is not that very important, because most of them they do maternal health, child health. There is a lot of activities happening, workshops and stuff. (27RMW)*
HR policies not conducive to continuity and stability in surgical teams	*But rotation...obviously it's like you are a jack of all trades and you are a master of none. (27RMW)*
HR failure to provide cover for staffing gaps	*There are two [NPCs}, one is at school he is doing basic surgery. So when that guy is gone to school we only have one, so it's difficult. (09DMW)*
INTRINSIC FACTORS	
Lack of confidence and skills	*Most of them, they don't have the confidence to tackle surgical issues. So they feel like if they mess up it would be on their necks. (27RMW)*
Lack of interest	*Currently I think we have some who can do [surgery], but they don't have an interest in that area. (12DMW)*

Divergence into other tasks	*There are some clinicians who are coordinators of TB, they are out maybe doing supervision, doing trainings. And sometimes there are some clinicians who are doing HIV training so they are not in the facilities. So these clinicians they forget about doing the surgeries, so they forget the skills. (10DMW)*
EMERGING DYNAMICS	
Delegation of surgical responsibilities to few more experienced clinicians	*There are nine clinicians, for example this man can only do caesarean sections, doesn't do other procedures. So he is expert only in caesarean sections but if a laparotomy comes he needs to call someone who is the one who knows how to do the laparotomy. […] we do refer unnecessarily because we depend on one man. (07DMW)*
Progressive disengagement from surgery	*Some people don't have confidence or they think this one who has a basis in surgery, he is the one who knows and we don't know. [...] They expect the one in surgery to be the one to take care of everything. (27RMW)*
